# Strongy Detect: Preliminary Validation of a Prototype Recombinant Ss-NIE/Ss-IR Based ELISA to Detect *Strongyloides stercoralis* Infection

**DOI:** 10.1371/journal.pntd.0010126

**Published:** 2022-01-25

**Authors:** William J. Sears, Thomas B. Nutman

**Affiliations:** Laboratory of Parasitic Diseases, National Institute of Allergy and Infectious Diseases, Bethesda, Maryland, United States of America; University of Passo Fundo: Universidade de Passo Fundo, BRAZIL

## Abstract

**Background:**

*Strongyloides stercoralis* (Ss) is the etiological agent of strongyloidiasis, a neglected tropical disease of global concern. Laboratory diagnosis of strongyloidiasis is most often based on detection of antibodies against antigens in an enzyme linked immunosorbent assay (ELISA). Herein, we report a preliminary validation study of newly developed IgG4- and/or IgG- based ELISAs to detect strongyloidiasis (Strongy Detect, InBios) incorporating a cocktail of 2 previously described recombinant antigens, Ss-NIE and Ss-IR.

**Methods:**

The sensitivity and specificity were determined by using the assay in 150 cryopreserved serum samples from humans known to be Ss infected (n = 74), helminth uninfected (n = 47), or infected with a helminth other than Ss [n = 29). The treatment associated dynamics of antibody detection were then assessed using 35 paired samples obtained before and after definitive therapy.

**Results:**

The IgG and IgG4 assays were 99% and 96% sensitive, respectively, and 99% and 100% specific, respectively. Neither the IgG or IgG4 assay showed cross reactions with sera from those infected with other helminths. Although ELISA values did decline post-treatment few returned to levels below the cutoff for infection.

**Conclusion:**

Strongy Detect is the most sensitive and specific commercialized immunoassay for detection of strongyloidiasis. The assay remains positive for greater than a year post-treatment.

## Author summary

*Strongyloides stercoralis* is a worm that infects people and some animals. It can live in its host for decades because it can continually produce more larvae that develop inside the body of its host. The initial infection usually occurs by larvae burrowing through the skin of a person. The growing larvae travel to the lungs where they are coughed up and swallowed. Then, in the intestine, they mature to adults capable of producing eggs. The eggs hatch before they can be passed out of the body in the stool. The newly hatched larvae burrow through the gut wall and repeat this internal cycle. To diagnose this infection, one strategy is detecting antibodies against these worms. Herein, we discuss a prototype ELISA assay using recombinant *Strongyloides stercoralis* proteins. The test, called Strongy Detect, did well at detecting antibodies in patients known to have the infection and at calling samples negative when the patients were uninfected. The antibodies were detectable more than a year after patients were treated for the disease. If further validation studies confirm the utility of this assay, it will be an important addition to the armamentarium of tests to diagnose *Strongyloides stercoralis* infection.

## Introduction

*Strongyloides stercoralis* (Ss) is a globally distributed nematode and the major etiologic agent of strongyloidiasis, a neglected tropical disease [1,2]. The detection of antibodies against Ss has proven to be a robust diagnostic method and, as such, has become standard of care given the relatively poor sensitivity of parasitological methods [3–6]. A widely applied method employs soluble extracts of adult worms or larvae from either *S*. *stercoralis* or *S*. *ratti* [7]. These so-called crude-antigen based immunoassays are available commercially (IVD Research, California; Bordier, Switzerland), and in in-house “research only” assays, including those performed at the CDC (personal communication) and an indirect immunofluorescent antibody test (IFA) [8,9]. While crude antigen-based immunoassays have proven sensitive, the specificity is suboptimal due to cross reactions with other helminths [10,11]. Efforts to improve Ss immunoassays led to the discovery and recombinant production of the Ss-NIE and Ss-IR antigens [12–14].

Ss-NIE/Ss-IR recombinant antigens have been successfully adapted to various diagnostic platforms including immunoblots, enzyme linked immunosorbent assays (ELISA), and Luciferase Immunoprecipitation Assay (LIPS) [15,16]. Although LIPS is a highly effective system, clinical laboratories have more familiarity with performing ELISAs. Recently, Inbios (Seattle, WA) undertook the development of a commercial recombinant antigen-based ELISA to detect Ss infection. Termed Strongy Detect, these prototype assays detect either IgG or IgG4 to a cocktail of 2 Strongyloides-specific recombinant proteins Ss-NIE and Ss-IR. A recently published study evaluated the performance characteristics of this assay using a repository of sera [17]. Herein, we describe the performance of both the IgG and IgG4 detection-based ELISA assays using well-characterized sera from the biorepository at the Laboratory of Parasitic Disease (LPD) at the National Institute of Allergy and Infectious Disease (NIAID).

## Methods

### Ethics statement

This study was approved by the Institutional Review Board at the National Institutes of Health (NIH) (Protocol 09-I-N07) and written informed consent was obtained from all patients or from the parent/guardian of patients. The involvement of the LPD in Ss-NIE (patent holder) has not influenced the selection of sera or the conduction of the study.

### Patient samples

The sera used were obtained from patients and healthy volunteers seen as part of ongoing clinical protocols in the Laboratory of Parasitic Diseases. Informed written consent was obtained from all subjects. Sera were from patients aged 3 to 86 (Table A in S1 Text). The group of Ss positive patients (n = 74) all had documented Ss larvae in their stool. One portion (n = 47) of the Ss negative group were healthy donors without epidemiologic risk of Ss infection. The second portion (n = 29) of Ss negative patients were documented to be infected with helminths other than Ss (*Wuchereria bancrofti*, n = 1; *Schistosoma* spp, n = 1; *Trichuris trichiura*, n = 1; *Toxocara canis*, n = 1; *Onchocerca volvulus*, n = 1; *Loa loa*, n = 22; or *Onchocerca volvulus*/*Loa loa* co-infection, n = 2). The sera used to compare the pre- and post-treatment ELISA values were taken from patients with documented larvae in stool, documented treatment date, and documented negative stool exam following treatment (either a one-time dose of 200 μg/kg Ivermectin or 200μg/kg Ivermectin given daily for two consecutive days, or 25mg/kg of thiabendazole given twice daily for 2 days or 25mg/kg thiabendazole twice daily for 3 days) as previously documented [18,19].

### ELISA assays

All assays were performed according to manufacturer protocol with the products provided in the Strongy Detect kit with negative and positive controls. Briefly, sera were diluted (1:25 for IgG4 assay; 1:100 for IgG assay) and then 100 microliters of each sample was added to a well in a 96 well plate and incubated for 30 minutes followed by a wash. All samples were run in duplicate. Following a 30 minute incubation and wash, a secondary antibody (horse radish peroxidase (HRP) conjugated anti-human-IgG or HRP conjugated anti-human-IgG4) was added for an additional 30 minutes. The plates were developed for 10 minutes with a solution containing tetramethylbenzidine (TMB) and hydrogen peroxide. A stop solution containing sulfuric acid was then applied and the assays were read using a SpectraMax spectrometer (Molecular Devices, San Jose, California).

Data were analyzed both by ODs as well as by signal/noise (S/N) calculations, the latter obtained by dividing the average OD for the sample/average OD of the plate blanks.

### Statistical analyses

Unless otherwise stated, geometric means were used as measures of central tendency. The cutoffs were determined by maximizing sensitivity and specificity based on receiver operator curves with confidence intervals calculated using the Wilson/Brown method [20]. Pre-and post-treatment antibody levels were compared using the Wilcoxon matched pairs signed rank test. All statistical analyses were performed in Prism Version 9.2.0 (GraphPad, San Diego, California).

## Results

**Fig 1:**
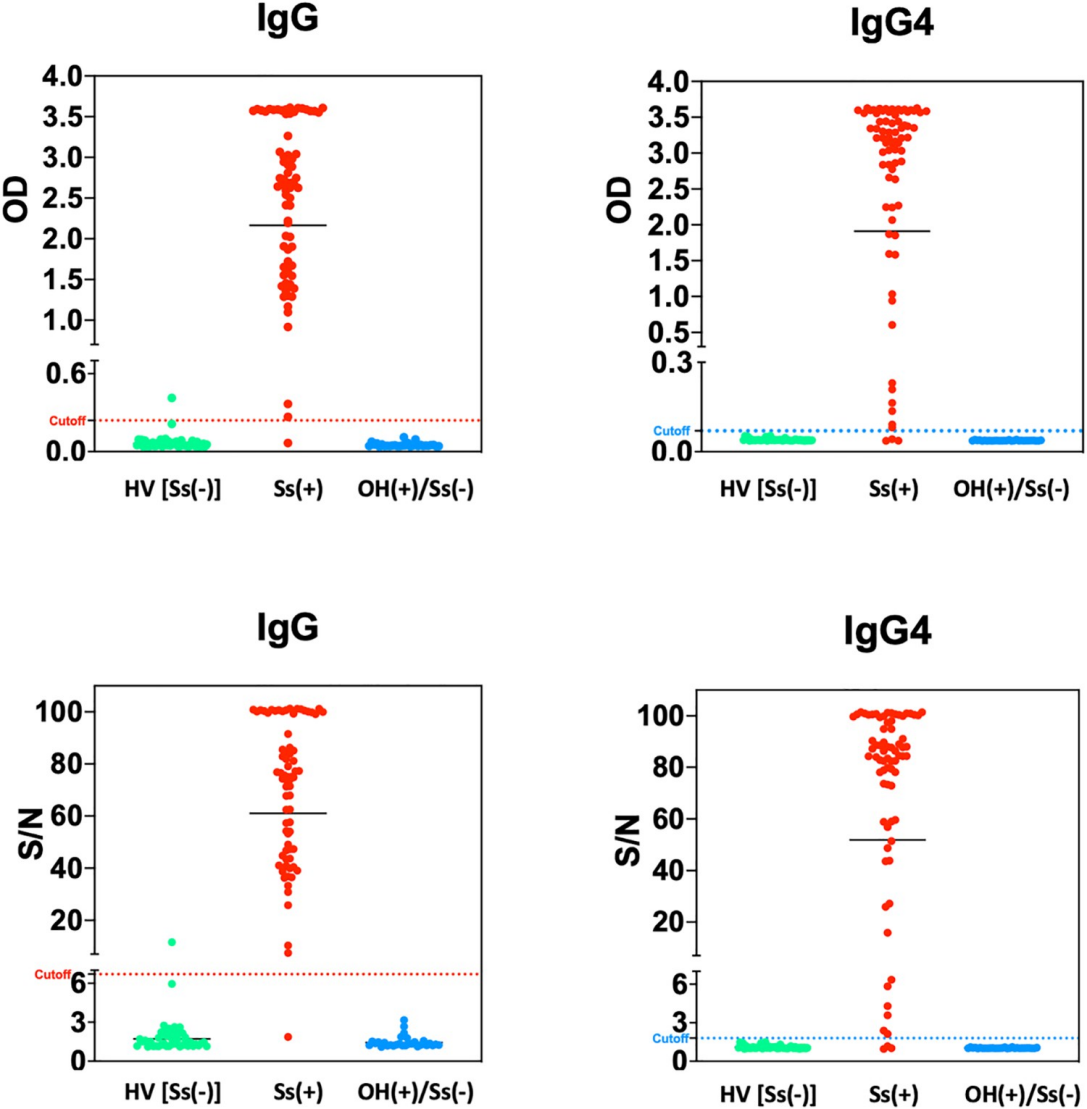
A comparison between the performance of the IgG and IgG4 based ELISA assays using raw optical density (OD) measurements and signal-to-noise ratio (S/N) when applied to sera from healthy controls, *Strongyloides stercoralis* (Ss) infected (documented larvae in stool), and those with helminth infections other than Ss. The dashed line represents the cutoff of the respective tests calculated from the cumulative data. The data points above the dotted line are considered positive and those below are considered negative. (HV = Healthy Volunteers; SS+ = Ss larvae in stool; OH = patients infected with helminths other than Ss).

The IgG and IgG4 capture assays were run in parallel for all 74 pre-treatment Ss stool positive samples and the 76 Ss-uninfected samples (47 = HV; 29 = other helminths). Data were analyzed both using optical densities (ODs) and signal/noise (S/N) ratios. Appropriate positive and negative control values were obtained for each plate. The receiver operator curves (ROC; Fig A in S1 Text) suggested optimal cutoffs of 6.7 for IgG S/N, 1.8 for IgG4 S/N, 0.24 for IgG ODs and 0.07 for IgG4 ODs (Table 1). These assays have a high degree of separation between true positives (Ss infection) and negatives (Ss-uninfected) (Fig 1). Importantly, there were no false positives in sera from patients infected with helminths other than Ss (Fig 1). The average value of the positive controls were 2.7, 71.7, 1.5, and 78 for the IgG OD, IgG S/N, IgG4 OD, and IgG4 S/N, respectively. The average value of the negative controls were 0.09, 2.5, 0.04, 1.08 for IgG OD, IgG S/N, IgG4 OD, and IgG4 S/N, respectively.

**Table 1:** The sensitivity and specificity of the optimal cutoffs for signal-to-noise (S/N) and optical density (OD) was calculated for the IgG and IgG4 assays. (SN = Signal/Noise Ratio, OD = Optical Density).

	Sensitivity (%)	Specificity (%)	Cutoff
**SNIgG**	**98.6**	**98.7**	**6.7**
**SNIgG4**	**95.9**	**100**	**1.8**
**ODIgG**	**98.6**	**98.7**	**0.24**
**ODIgG4**	**95.9**	**100**	**0.07**

**Fig 2:**
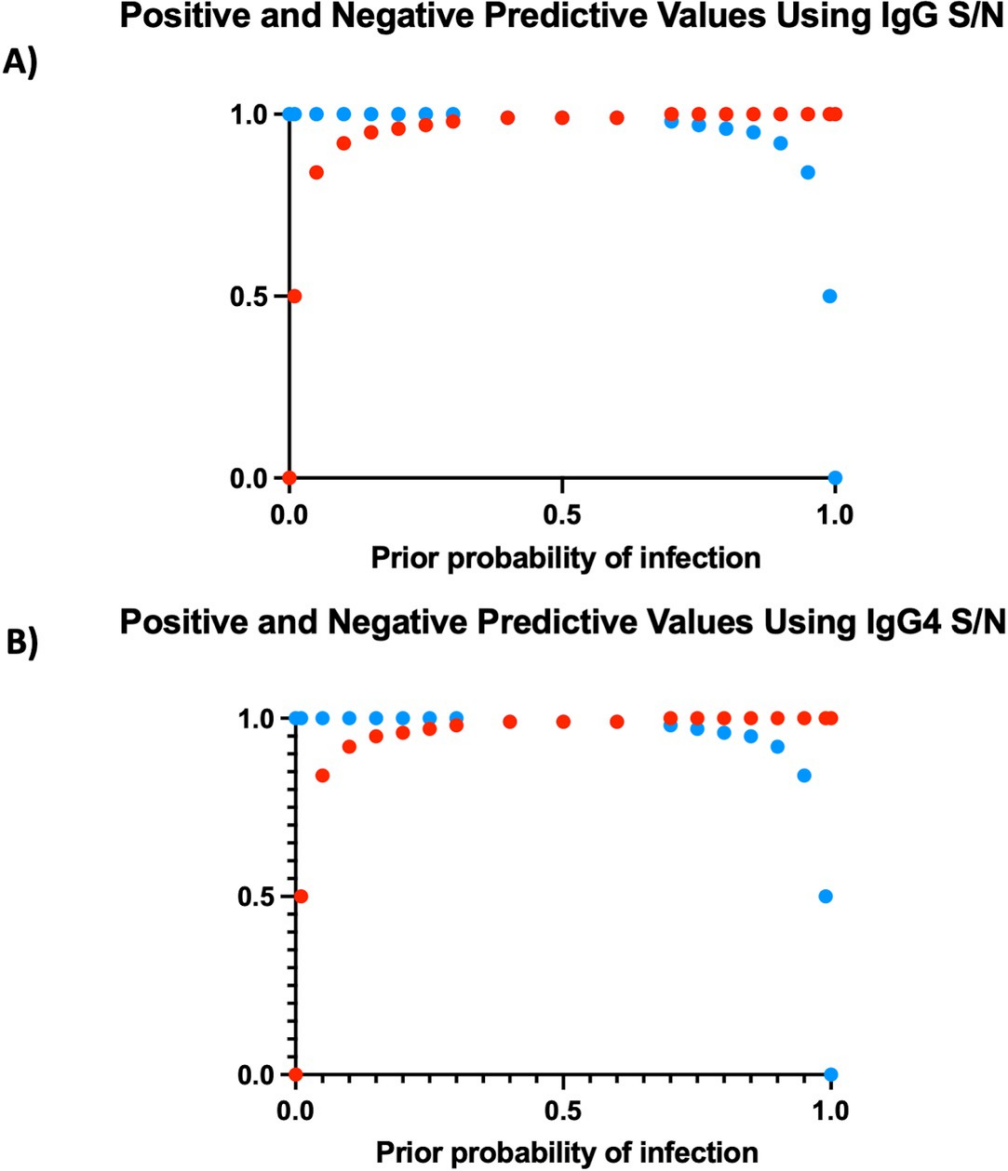
A comparison between the optimal cutoffs of the IgG and IgG4 based assays and their predicted positive (PPV) and negative predictive values (NPV) for a given prevalence. Using the calculated cutoff value, a plot of the positive and negative predictive values for a given prior probability of infection was calculated using IgG S/N (A) and IgG4 S/N (B). (Blue = NPV; Red = PPV).

Using the cutoffs based on the ROCs, the sensitivity and specificity calculated by S/N were equal to sensitivity and specificity calculated by OD for each respective assay (Table 1). Using the ODs, the sensitivities of the IgG and IgG4 assays were 98.6% and 95.9%, respectively. The specificities, based on S/N ratios were 98.6% (for IgG) and 100% (for IgG4). We next used these sensitivities and specificities to calculate the positive and negative predictive values expected across the range of given prior probabilities of infection (Fig 2A and 2B). As can be seen, both the IgG- and IgG4-based assays would be expected to perform well across the spectrum of prior probabilities.

The temporal dynamics of the antibody response as measured by these ELISAs were assessed in 35 sets of paired samples taken prior to definitive treatment for strongyloidiasis and at timepoints subsequent to treatment. Although a statistically significant decline in most patients’ antibody levels was observed (p = .001) over one year, few patients’ levels declined to below the positive cutoff at any timepoint including those sampled at greater than 1 year.

## Discussion

*Strongyloides stercoralis* has a global distribution albeit with varying degrees of geographic endemicity and frequent co-endemicity with other helminths [21]. The adaptation of the Ss-NIE and Ss-IR recombinant antigens into various platforms has consistently improved the ability to accurately diagnose strongyloidiasis [10,16,18,22]. ELISAs are often employed in clinical laboratories due to ease of use, adaptability into existing workflows, and rapid, quantifiable results. However, until recently, there existed no commercialized ELISA incorporating Ss-NIE/Ss-IR. The Strongy Detect assay preliminarily evaluated herein holds promise to bridge the improved Ss diagnostic performance provided by recombinant technology with the many clinical labs equipped to perform ELISAs.

Using stringent criteria, and regardless of whether S/N or OD values were used, both the IgG and IgG4 ELISAs were highly sensitive and specific, consistent with other methodologies incorporating Ss-NIE/Ss-IR. The highly specific nature of this assay in patients co-infected with other helminths suggests a potential improvement over the cross-reactivity observed in the crude-antigen based ELISA. An improved specificity and, thus, positive predictive value would improve not only clinical diagnosis but also refine our epidemiological understanding of disease burden, especially in areas where the geographic range of helminths overlap.

IgG4 was targeted in addition to IgG as previous studies have shown that IgG4-based assays often have increased specificity over IgG-based assays [23]. IgG4 is the isotype employed in a number of antibody detection immunoassays for a number of helminths including those against *Loa loa* (Ll-SXP 1), *Wuchereria bancrofti* (Wb123), and *Onchocerca volvulus* (OV16) [23,24–26]. Interestingly, the data presented herein suggest that the sensitivity and specificity of the IgG- and IgG4-based Strongy Detect prototype ELISAs are largely equivalent.

A previous study evaluated this assay using a retrospective cohort and associated biorepository [17]. When using similar criteria to define true positivity and negativity, the IgG and IgG4 based assays yielded sensitivities of 92% and 81% and specificities of 91% and 94%, respectively. When more inclusive criteria of true positivity/negativity were applied, the “Composite Reference Standard” (CRS) as the authors termed it, the sensitivities were 78% and 75% while specificities were 98% and 91% for IgG and IgG4, respectively. The differences in results between the previous study and those contained herein are likely due to differing definitions of true positivity and differing derived cutoff values. It will be important for laboratories to assess this assay in the context of their own patient populations, equipment, and definitions.

**Fig 3:**
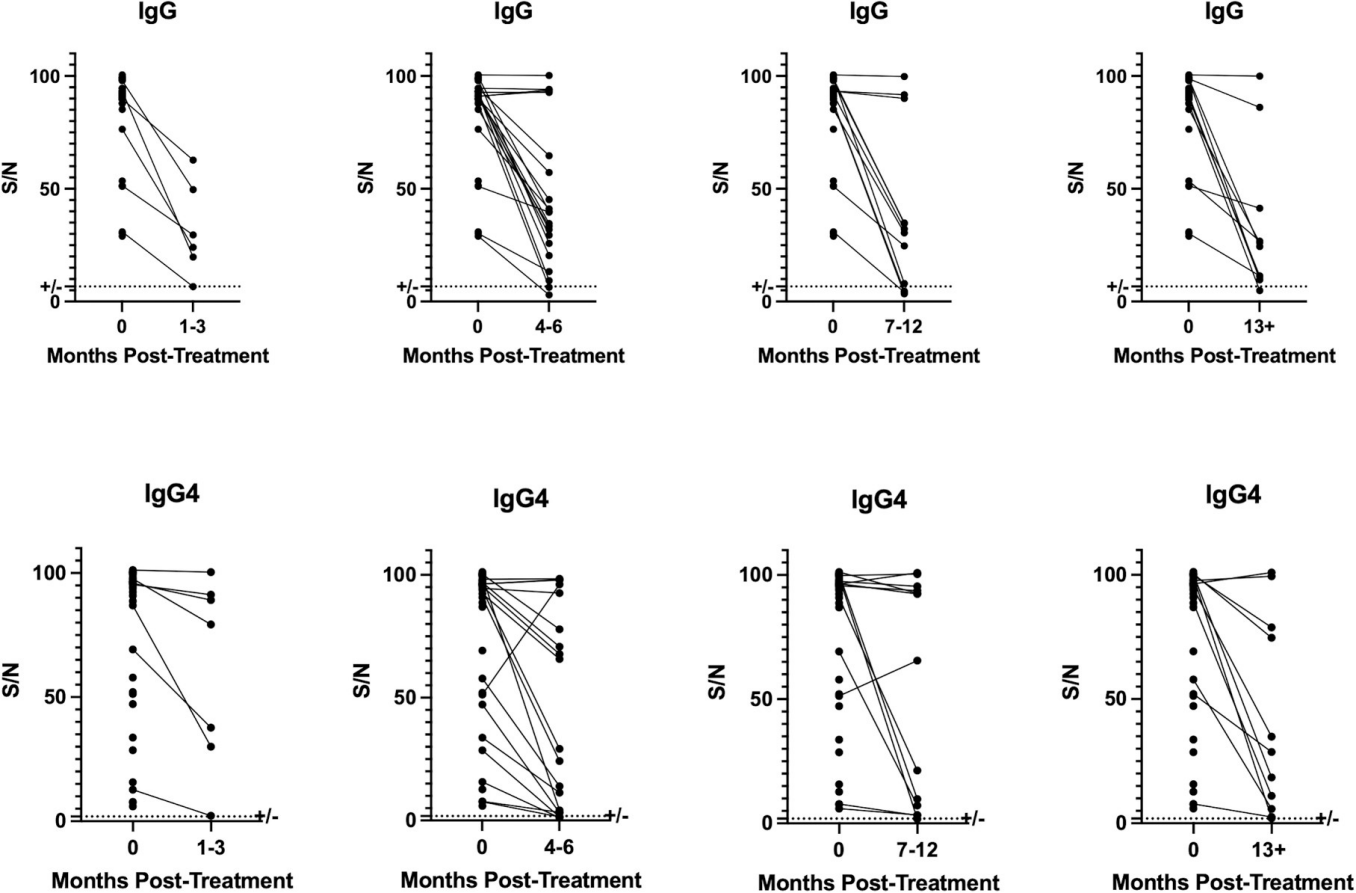
The values of IgG and IgG4 serum based ELISA assays before treatment was compared to post-treatment serum ELISA values obtained at 1–3 months, 4–6 months, 7–12 months or greater than a year post-treatment. The dotted line represents the calculated cutoffs for the respective assays.

Although the data support improvement in diagnostic capacity, both the IgG and IgG4 response against Ss-NIE and Ss-IR appear to persist above a positive cutoff longer than a year after a patient receives definitive therapy. (Fig 3) Therefore, this assay will not likely be helpful in determining cure. This result is consistent with previous studies showing Ss-IR to be highly immunogenic. Indeed, Ss-IR was used to induce a protective immune response against Ss in a murine model of Ss [27].

This study is limited by a relatively small sample size. Additionally, the lack of an internationally recognized gold standard for proof of cure complicates the pretreatment/posttreatment intrasubject comparison. We attempted to compensate for this by applying stringent selection criteria and only selecting patients who were documented to have had received therapy and had no larvae detected on subsequent stool exams. Also, cutoff values as determined by our patient population may not be applicable to all geographic areas and patient populations.

In conclusion, the data presented suggest that the Inbios Strongy Detect ELISA incorporating a cocktail of Ss-NIE and Ss-IR as capture antigens has improved sensitivity and specificity over the currently employed ELISAs to detect Ss. In comparing the IgG with IgG4 based assays, the results were equivalently highly sensitive and specific. While potentially improving the ability to diagnose strongyloidiasis, the test does not seem to be appropriate for test of cure due to continued presence of anti-Ss-NIE and anti-Ss-IR antibodies following definitive therapy. Taken together, these data support the utility of Strongy Detect ELISA as potentially being a sensitive and specific assay for the detection of Ss infection.

## Supporting information

S1 Text **Supporting Information Fig A.** The ROC curves for the respective assays were used to determine the cutoff values for each ELISA using both optical density and signal/noise ratio. **Fig B.** The pre/post treatment optical density values obtained by either an IgG- or IgG4- based ELISA at the given duration following therapy for *Strongyloides stercoralis*. **Table A.** Demographics of patients included in the study.
(DOCX)
