# Supplementary material for: Strongy Detect: Preliminary Validation of a Prototype Recombinant Ss-NIE/Ss-IR Based ELISA to Detect Strongyloides stercoralis Infection
Source: PLoS Negl Trop Dis. 2022 Jan 25;16(1):e0010126. doi: 10.1371/journal.pntd.0010126 (PMC8789141; doi:10.1371/journal.pntd.0010126)
Supplement: S1 Text — Supporting Information Fig A. The ROC curves for the respective assays were used to determine the cutoff values for each ELISA using both optical density and signal/noise ratio. Fig B. The pre/post treatment optical density values obtained by either an IgG- or IgG4- based ELISA at the given duration following therapy for Strongyloides stercoralis. Table A. Demographics of patients included in the study. (DOCX) [file pntd.0010126.s001.docx]

**Supporting Information**

**Fig A**: The ROC curves for the respective assays were used to determine the cutoff values for each ELISA using both optical density and signal/noise ratio.

**Fig B**: The pre/post treatment optical density values obtained by either an IgG or IgG4 based ELISA at the given duration following therapy for *Strongyloides stercoralis.*

**Table A**: Demographics of patients included in the study.

**A**

**B**

**Table A**

| **Group** | **Median Age (range)** | **N (%F)*** |
| --- | --- | --- |
|  |  |  |
| **Ss+** | **38 (3-86)** | **109 (64% )** |
| **Ss-** | **35 (4-69)** | **47 (50%)** |
| **OtherHelminth+ Ss-** | **27 (10-61)** | **29 (54%)** |

*For all groups some missing values
